# Association between pro-inflammatory diet and fecal incontinence: a large population-based study

**DOI:** 10.3389/fnut.2025.1547406

**Published:** 2025-05-22

**Authors:** Haiyang Wang, Zihan Liu, Xingfu Lu, Enyu Luan, Tingting Dong, Can Li, Yanni Lv, Erkang Wu, Tao Shen

**Affiliations:** ^1^Anhui Provincial Key Laboratory of Molecular Enzymology and Mechanism of Major Metabolic Diseases, College of Life Sciences, Anhui Normal University, Wuhu, Anhui, China; ^2^Auhui Provincial Engineering Research Centre for Molecular Detection and Diagnostics, College of Life Sciences, Anhui Normal University, Wuhu, Anhui, China

**Keywords:** fecal incontinence, dietary inflammatory index, pro-inflammatory diet, NHANES, machine learning, risk prediction, inflammation

## Abstract

**Background:**

It is widely acknowledged that dietary habits play a pivotal role in maintaining optimal intestinal health. Fecal incontinence (FI) is a distressing and often stigmatized inflammatory ailment with a strong correlation to a multitude of intestinal disorders. However, the associations between diets and FI are largely unknown.

**Methods:**

In this study, we collected cross-sectional data from 31,034 participants enrolled in the National Health and Nutrition Examination Survey (NHANES). To investigate the relationship between pro-inflammation diet and the prevalence of FI, dietary inflammatory index (DII) was calculated, and participants were categorized into three tertiles. Meanwhile, we identified key dietary factors for FI using multiple machine learning algorithms. Finally, we assessed the mediation role of inflammatory indicators on the association of key dietary factors with FI through mediation analysis.

**Results:**

After adjustment for potential confounding variables, our results showed the highest tertile exhibited dramatically increasing prevalence of FI compared to the lower tertile (OR 1.27, 95% CI 1.06–1.53), suggesting a positive association between DII and FI. We also identified total saturated fat, polyunsaturated fatty acid, vitamin A, *β* carotene, vitamin B2, and iron are the primary dietary factors associated with FI. Based on these dietary factors, we developed a novel FI risk prediction model. The receiver operating characteristic (ROC) analyses conducted on both the training and independent validation cohorts demonstrate favorable predictive performance for FI in nearly 10,000 participants. Moreover, our findings indicate that consumption of these key dietary factors can trigger an intestinal inflammatory response by mediating neutrophils and lymphocytes, which in turn contributes to the development of FI.

**Conclusion:**

In conclusion, this study not only elucidates the fundamental roles of pro-inflammatory diet in inducing intestinal inflammation and eventually in resulting FI, but also provides a FI risk prediction tool based on dietary factors, which may prove beneficial in the clinical diagnosis of FI.

## Introduction

Fecal incontinence (FI) is an unpleasant and frequently stigmatized disorder that usually entails trouble controlling the evacuation of bowel contents or repeated, involuntary evacuation of stool (solid, liquid, or mucus) (1, 2). Previous studies indicate that the prevalence of fecal incontinence (FI) among residents in nursing homes within Leicester, England, is slightly below 10% (3, 4). Individuals with FI frequently experience social isolation, melancholy, poor self-image, unemployment, and shame (2). These conditions create a variety of social, psychological, and physical difficulties that significantly lower their quality of life (5). Due to the devastating impact FI can have on daily life, such as loss of confidence, self-esteem, modesty and composure, many FI patients avoid discussing their illness with friends, family, and medical professionals. In certain cases, they even avoid them altogether (1, 6). Lack of knowledge about FI, which results in patients’ inability to adequately characterize their illness when not given specifically defined questions, is undoubtedly another important contributing cause to this phenomena (7). An Internet-based survey found that only 30% of nearly 1,100 community-dwelling women with FI had heard of the term “fecal incontinence,” with the majority (70%) preferring the term “accidental leakage of bowel” to describe their condition (8). In addition, people are rarely screened for FI during routine health care, as shown in a previous study where only 17% of women with FI receiving benign gynecological care were asked about symptoms by their health care provider, and only 2.7% had a medical diagnosis of FI (9, 10). The severe social stigma of FI and the lack of consensus on screening for FI results in significant under-reporting of FI conditions, which can lead to further mismanagement of FI (7).

Recent epidemiological studies have found that intestinal disorders such as diarrhea and bowel urgency may be associated with FI, and that dietary intake leading to the symptoms of these intestinal disorders may be an important risk factor for the development of FI (11). For instance, high-fat diets have been shown to exacerbate FI symptoms through dual pathways, including inducing hard stools *via* excessive fluid absorption (12, 13), and impairing gastrointestinal motility by compromising enteric nervous system function (12, 14). Conversely, dietary interventions demonstrate therapeutic potential, with psyllium fiber supplementation showing protective effects (15), while low-fermentable oligosaccharides, disaccharides, monosaccharides, and polyols (FODMAP) diets achieving symptom alleviation (16, 17). However, the existing evidence remains fragmented with limited mechanistic exploration, particularly regarding nutrient-gut axis interactions.

Currently, it is thought that diet may be an important factor in regulating systemic inflammation (18). With the emergence of diverse dietary cultures, it has led to the formation of various dietary patterns, including those that are pro-inflammatory, which, to a certain extent, can contribute to the development of chronic inflammatory diseases, including intestinal inflammation (19–21). For example, the Western dietary pattern—characterized by high intake of processed foods rich in saturated fats and refined sugars combined with low dietary fiber—has been shown to induce gut dysbiosis and increase pro-inflammatory bacterial populations, contributing to systemic inflammation (22, 23). The Empirical Dietary Inflammation Pattern (EDIP), a hypothesis-driven model, quantifies dietary inflammatory potential by correlating food intake with inflammatory biomarkers such as IL-6, CRP, and TNF-*α*, where higher EDIP scores reflect stronger pro-inflammatory effects (24, 25). At the nutrient level, evidence indicates that high-carbohydrate diets exacerbate inflammation through insulin resistance and glucose toxicity (26), while excessive cholesterol intake promotes oxidative stress and impairs high-density lipoprotein cholesterol function (27). FI, as a manifestation of intestinal inflammation (5), might be affected by the inflammatory diet.

In order to fully and accurately quantify the inflammatory capacity of the diet, Shivappa et al. developed the dietary inflammatory index (DII) (28). The 25 dietary components in DII derived from NHANES data, which includes energy, macronutrients (protein, carbohydrate, total fat), fatty acid subtypes (saturated, monounsaturated, polyunsaturated), cholesterol, dietary fiber, *β*-carotene, niacin, and micronutrients such as vitamins (A, B1/B2/B6/B12, C, E, folate), minerals (magnesium, iron, zinc, selenium), along with caffeine and alcohol were reported to measure the inflammatory potential of one’s diet (29). The DII scoring system assigns “+1” to components positively associated with pro-inflammatory biomarkers (e.g., CRP, TNF-*α*, IL-1β, IL-6) or negatively linked to anti-inflammatory cytokines (IL-4, IL-10), and “-1” for those showing the opposite effects. Specific components demonstrate distinct inflammatory roles: saturated fats may synergize with environmental toxins like cadmium to induce macrophage-driven inflammation (30), while vitamin C acts as an antioxidant through electron donation (31), and vitamin A modulates T-cell differentiation to maintain immune balance (32). However, the inflammatory effects of certain nutrients (e.g., niacin, caffeine, magnesium, etc.) appear context-dependent, with evidence suggesting dose-dependent duality or interactions with other dietary factors (33–35). Given DII has been widely and strongly associated with gastrointestinal disorders such as ulcerative colitis, crohn’s disease, and others (36–39), the index may serve as a tool for investigating FI pathogenesis.

The purpose of this study was to ascertain the relationship between inflammatory diet and FI, therefore cross-sectional data from 31,034 participants across 2005 to 2010 were retrieved from the NHANES database and the DII was utilized to assess levels of diet-induced inflammation. Our results not only discovered a nonlinear positive connection between DII and the incidence of FI, but also identified total saturated fat, polyunsaturated fatty acid, vitamin A, *β* carotene, vitamin B2, and iron as key dietary factors contributing to the development of FI. Based on the intakes of these key dietary factors, the occurrence of FI could be well predicted. Mechanistically, the present study found intaking the indicated key dietary factors activate the intestinal inflammatory response by mediating neutrophils and lymphocytes, which in turn contribute to the development of FI. In conclusion, the present study not only revealed the fundamental roles of pro-inflammatory diets in inducing FI, but also provides an adjunct tool for FI’s early screening, which will expand the current understanding of diet and health.

## Materials and methods

### Study population

**Figure 1:**
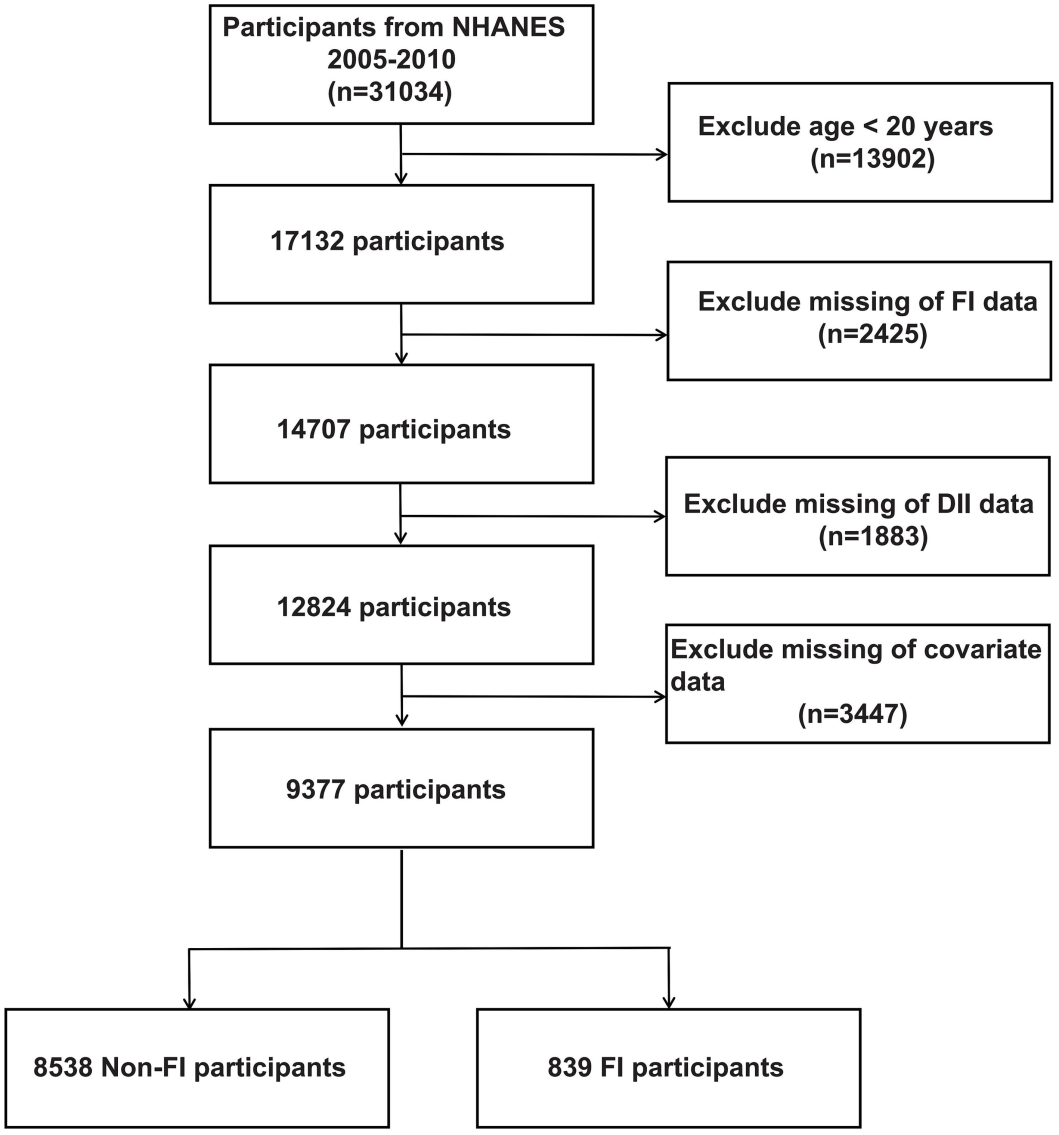
The flow chart of participant recruitment from NHANES 2005–2010.

NHANES is a comprehensive cross-sectional study administered by the National Center for Health Statistics. Its purpose is to collect important data on the health status of individuals by conducting a series of interviews, physical assessments, and laboratory tests. To ensure an accurate representation of the overall U.S. population, the survey utilizes advanced multistage sampling techniques. The study protocol adhered to the Declaration of Helsinki and ethical approval was obtained from the National Health and Wellness Council’s Research Ethics Review Board, and all adult participants provided written informed consent. Specifically, the present study retrieved cross-sectional data from the NHANES database for 31,034 participants across 2005 to 2010 based on the principle of being able to recognize the characteristics of FI patients. Exclusion criteria were as follows: (a) exclusion of participants younger than 20 years of age (*n* = 13,902), (b) participants with missing FI data (*n* = 2,425), (c) participants without dietary data used to calculate the DII (*n* = 1883), (d) participants with missing covariate data (*n* = 3,447), resulting in 9377 participants included in the analyses, which included 8,538 Non-FI participants and 839 FI participants, as shown in Figure 1.

### Calculation of DII

As previously reported (40–43), the DII developed by Shivappa et al. was used to measure the inflammatory potential of different dietary patterns (28). It contains 25 dietary components from NHANES, including energy (kcal), protein (g), carbohydrate (g), dietary fiber (g), total fat (g), total saturated fat (g), monounsaturated fatty acid (g), polyunsaturated fatty acid (g), cholesterol (mg), vitamin E (mg), vitamin A (mcg), beta carotene (mcg), vitamin B1 (mg), vitamin B2 (mg), niacin (mg), vitamin B6 (mg), total folate (mcg), vitamin B12 (mcg), vitamin C (mg), magnesium (mg), iron (mg), zinc (mg), selenium (mcg), caffeine (mg) and alcohol (g). Standardized means and standard deviations for all DII food parameters were provided in the global database. A scoring system was established to ensure the comprehensiveness and accuracy of the survey. A score of “+1” was given if the dietary component increased the levels of CRP, TNF-a, IL-1b, and IL-6 or decreased the levels of IL-4 and IL-10. Conversely, a score of “-1” is given if the dietary component decreases levels of CRP, TNF-a, IL-1b, and IL-6, or increases levels of IL-4 and IL-10. An elevated DII score would indicate the consumption of a diet that triggers inflammation, while a lower score would indicate the consumption of an anti-inflammatory diet. This comprehensive approach ensures an accurate and representative assessment of the inflammatory potential of an individual’s diet. Specifically, the mean DII score for the overall study population was 1.35. From tertile 1 (T1) to tertile 3 (T3), DII scores ranged from-4.54 to 0.79, 0.79 to 2.22, and 2.22 to 4.49, respectively.

### Assessment of FI

Bowel Health Questionnaire (BHQ) data from three NHANES cohorts (2005–2006, 2007–2008, 2009–2010) were used in this study to determine whether participants had FI. The present study selected these three cycles because, during this period, the BHQ meticulously recorded information pertinent to FI. The assessment of FI was primarily based on Rockwood’s FI severity index, which is comprised of four symptoms: mucus, liquid, solid, and gas stools incontinence (44). Frequency is determined by how often it occurs: never, 1–3 times per month, once per week, 2 or more times per week, once per day, 2 or more times per day. FI is defined as any involuntary loss of mucus, liquid, or solid feces in the past 30 days, this definition of FI excludes gas (45).

### Covariates

The present study collected a number of demographic factors of the participants based on previous studies and clinical practice, i.e., multiple covariates were considered, including socio-demographic factors (Gender, Age, Race), lifestyle factors (Smoked, Alcohol, BMI) and co-morbidities (Hypertension, Arthritis, Liver Disease, Cancer, Asthma) (46–50). Among these, gender was categorized into two groups (male/female), age was categorized into three groups (20–40 year, 41–60 year, ≥60 year), race was categorized into five groups (Mexican American/Other Hispanic/Non-Hispanic White/Non-Hispanic Black/Other Race), smoking status was categorized into two groups (yes/no) based on the answer to the question “Have smoked at least 100 cigarettes in your life,” drinking status was categorized into two groups (yes/no) based on the answer to the question “Have had at least 12 alcoholic drinks per year,” BMI was categorized into four groups (underweight, < 18.5 kg/m^2^; normal weight, 18.5–24.9 kg/m^2^; overweight, 25–29.9 kg/m^2^; obese, ≥30 kg/m^2^) (51–53), hypertension was categorized into two groups (yes/no) based on responses to the question “Has a doctor told you that you have high blood pressure,” arthritis was categorized into two groups (yes/no) based on responses to the question “Has a doctor told you that you have arthritis,” liver Disease was divided into two groups (yes/no) according to the question “Has a doctor told you that you have liver disease,” cancer was divided into two groups (yes/no) according to the question “Has a doctor told you that you have cancer,” asthma was categorized into two groups (yes/no) based on responses to the question “Has a doctor told you that you have asthma.”

### Identifying key dietary factors using multiple machine learning algorithms

To screen the key dietary factors from the 25 types of dietary components in DII, the present study employed 13 machine learning methods packaged into 93 algorithmic frameworks to identify the key dietary factors that contribute to FI (54, 55). The machine learning methods includes Logistic Regression (LR), Linear discriminant analysis (LDA), Quadratic discriminant analysis (QDA), k-Nearest Neighbor (KNN), Decision tree (DT), Random forest (RF), Extreme Gradient Boosting (XGBoost), ridge regression (RR), Lasso regression (Lasso), Elastic Net regression (ENR), Support vector machine (SVM), Grandient Boosting Machine (GBM), and Naive Bayesian algorithm (NaiveBayes). These algorithms play a fundamental role in the initial stages of key dietary screening and work in concert with other algorithms. Both individual algorithms and their combinations contribute to this comprehensive framework. The 2005–2006 dataset was used for variable selection and model construction. Ninety three permutations of the indicated 13 algorithms were evaluated in a 10-fold cross-validation framework, and all constructed models were validated in the 2007–2008 datasets. The C-index for each model based on the two cycle datasets (2005–2006, 2007–2008) from the NHANES database was calculated. We then ranked the predictive performance of the models based on the average C-index and selected the combination of algorithms with robust performance and clinical translational significance, i.e., the algorithm with the highest average C-index across the two cycle datasets was considered as optimal methods for screening key dietary factors.

### Construction of FI risk prediction models based on key dietary factors

Participants retrieved from the NHANES database in 2005–2008 were used as the training cohort for the construction of FI risk prediction model, while participants from NHANES 2009–2010 were applied as the validation cohort. Based on the identified 6 key dietary factors and the basic characteristics of these participants (gender, age, and race), R packages “rms” and “nomogramFormula” were applied to construct and validate the risk prediction model for FI. Additionally, ROC curves were used to assess the performance of the model to further ensure the reliability of the constructed risk model.

### Statistical analysis

All data in the present study were analyzed by R statistics and EmpowerStats software. Continuous variables were presented as weighted means and standard deviations (SD), while categorical variables were presented as frequencies and percentages. To examine differences in baseline characteristics between FI and Non-FI participants, continuous and categorical variables were compared using the student’s t-test and chi-square test, respectively. The DII score was included in the model as an independent variable, both in continuous and tertile forms [T1 (−4.54 to 0.79, *n* = 3,126), T2 (0.79–2.22, *n* = 3,125), T3 (2.22–4.49, *n* = 3,126)], to explore potential correlations with FI. A variety of multivariate logistic regression models-unadjusted and two adjusted models (Model I and Model II) were used to estimate odds ratios (OR) and 95% confidence intervals (CI) for the correlation between DII and FI. Model I adjusted for age, gender, and race, and Model II further adjusted for all covariates. In addition, subgroup analyses of all covariates were conducted to test for significant interactions between the indicated covariates and the relationships between DII and FI. Restricted cubic spline (RCS) curves are often used in statistical modeling to represent a nonlinear relationship between two variables. We further used the multivariate-corrected (model3) RCS curve to assess the relationship of DII and key dietary intake with the incidence of FI. “Mediation” package was utilized to perform Mediation analysis assessing the mediating effects of inflammatory indicators on the associations of key dietary factors with FI. We also included the systemic inflammation response index [SIRI, SIRI = (neutrophils * platelet)/lymphocyte] analysis, which measures the systemic inflammatory activity of an individual, indicating the balance between inflammation and immune response (56). In all analyses of this study, *p* < 0.05 was considered statistically significant.

## Results

### Participants’ baseline characteristics by tertile of DII

A total of 31,034 participants enrolled in NHANES from 2005 to 2010 were retrieved in this study, and Figure 1 summarizes the cohort selection process. After excluding participants younger than 20 years of age, participants with missing computed DII parameters, participants with missing defined FI parameters, and participants with missing covariates, a total of 9,377 participants, including 8,538 non-FI participants and 839 FI participants, were finally included in the following analyses. Table 1 shows the baseline characteristics of the participants included in the analyzed cohort by DII tertiles. Our result shows that the baseline characteristics, including age and gender, were evenly distributed, with 3,239 (34.54%) aged 20–40 years, 3,050 (32.53%) aged 40–60 years, and 3,088 (32.93%) aged >60 years. 4,660 (49.70%) males and 4,717 (50.30%) females present. Meanwhile, significant difference (−0.46 in T1, 1.53 in T2, and 2.97 in T3, *p* < 0.001) exists among the indicated three DII tertiles. Therefore, we further investigated the divergence in each dietary component involved in estimating DII among these groups. Our results revealed a lower intake of nearly all dietary components, with the exception of caffeine, in participants with high-DII rather than low-DII (Supplementary Table S1). Besides, according to our findings, differences among the DII tertiles for FI, gender, age, race, smoked, alcohol, BMI, hypertension, arthritis, and asthma were statistically significant. More specifically, people with higher DII scores are typically older, more likely to be female, frequent smokers, obese, and more susceptible to conditions like asthma, hypertension, and arthritis (Supplementary Figure S1). Notably, the prevalence of FI rose as DII scores rose, suggesting that elevated DII may be a possible causative component in the higher frequency of FI.

**Table 1:** Baseline characteristics of the study participants according to DII tertiles.

Variables	Total	DII-T1 (−4.54 to 0.79, *n* = 3,126)	DII-T2 (0.79–2.22, *n* = 3,125)	DII-T3 (2.22–4.49, *n* = 3,126)	*p*-value
DII	1.35 ± 1.57	−0.46 ± 0.96	1.53 ± 0.41	2.97 ± 0.50	<0.001
FI, n (%)					<0.001
Yes	839 (8.95%)	237 (7.58%)	280 (8.96%)	322 (10.30%)	
No	8,538 (91.05%)	2,889 (92.42%)	2,845 (91.04%)	2,804 (89.70%)	
Gender, n (%)					<0.001
Male	4,660 (49.70%)	1903 (60.88%)	1,570 (50.24%)	1,187 (37.97%)	
Female	4,717 (50.30%)	1,223 (39.12%)	1,555 (49.76%)	1939 (62.03%)	
Age, n (%)					0.007
20–40	3,239 (34.54%)	1,057 (33.81%)	1,109 (35.49%)	1,073 (34.33%)	
40–60	3,050 (32.53%)	1,065 (34.07%)	1,029 (32.93%)	956 (30.58%)	
≥60	3,088 (32.93%)	1,004 (32.12%)	987 (31.58%)	1,097 (35.09%)	
Race, n (%)					<0.001
Mexican American	1,688 (18.00%)	582 (18.62%)	605 (19.36%)	501 (16.03%)	
Other Hispanic	818 (8.72%)	240 (7.68%)	280 (8.96%)	298 (9.53%)	
Non-Hispanic White	4,703 (50.15%)	1742 (55.73%)	1,513 (48.42%)	1,448 (46.32%)	
Non-Hispanic Black	1815 (19.36%)	431 (13.79%)	597 (19.10%)	787 (25.18%)	
Other Race	353 (3.76%)	131 (4.19%)	130 (4.16%)	92 (2.94%)	
Smoked, n (%)					<0.001
Yes	4,468 (47.65%)	1,404 (44.91%)	1,434 (45.89%)	1,630 (52.14%)	
No	4,909 (52.35%)	1722 (55.09%)	1,691 (54.11%)	1,496 (47.86%)	
Alcohol, n (%)					<0.001
Yes	6,772 (72.22%)	2,437 (77.96%)	2,295 (73.44%)	2040 (65.26%)	
No	2,605 (27.78%)	689 (22.04%)	830 (26.56%)	1,086 (34.74%)	
BMI, n (%)					<0.001
Underweight	157 (1.67%)	47 (1.50%)	40 (1.28%)	70 (2.24%)	
Normal weight	2,703 (28.83%)	978 (31.29%)	894 (28.61%)	831 (26.58%)	
Overweight	3,316 (35.36%)	1,163 (37.20%)	1,117 (35.74%)	1,036 (33.14%)	
Obese	3,201 (34.14%)	938 (30.01%)	1,074 (34.37%)	1,189 (38.04%)	
Hypertension, n (%)					<0.001
Yes	3,006 (32.06%)	899 (28.76%)	987 (31.58%)	1,120 (35.83%)	
No	6,371 (67.94%)	2,227 (71.24%)	2,138 (68.42%)	2006 (64.17%)	
Arthritis, n (%)					<0.001
Yes	2,475 (26.39%)	751 (24.02%)	786 (25.15%)	938 (30.01%)	
No	6,902 (73.61%)	2,375 (75.98%)	2,339 (74.85%)	2,188 (69.99%)	
Liver disease, n (%)					0.687
Yes	298 (3.18%)	105 (3.36%)	93 (2.98%)	100 (3.20%)	
No	9,079 (96.82%)	3,021 (96.64%)	3,032 (97.02%)	3,026 (96.80%)	
Cancer, n (%)					0.083
Yes	899 (9.59%)	326 (10.43%)	274 (8.77%)	299 (9.56%)	
No	8,478 (90.41%)	2,800 (89.57%)	2,851 (91.23%)	2,827 (90.44%)	
Asthma, n (%)					0.001
Yes	1,214 (12.95%)	363 (11.61%)	394 (12.61%)	457 (14.62%)	
No	8,163 (87.05%)	2,763 (88.39%)	2,731 (87.39%)	2,669 (85.38%)	

Data are presented as mean ± SD or n (%). Continuous variables are presented as weighted mean (95% CI), and categorical variables are presented as unweighted frequencies or percentages (95% CI). CI, confidence interval, BMI, Body Mass Index.

### DII is nonlinearly and positively correlated to FI

**Figure 2:**
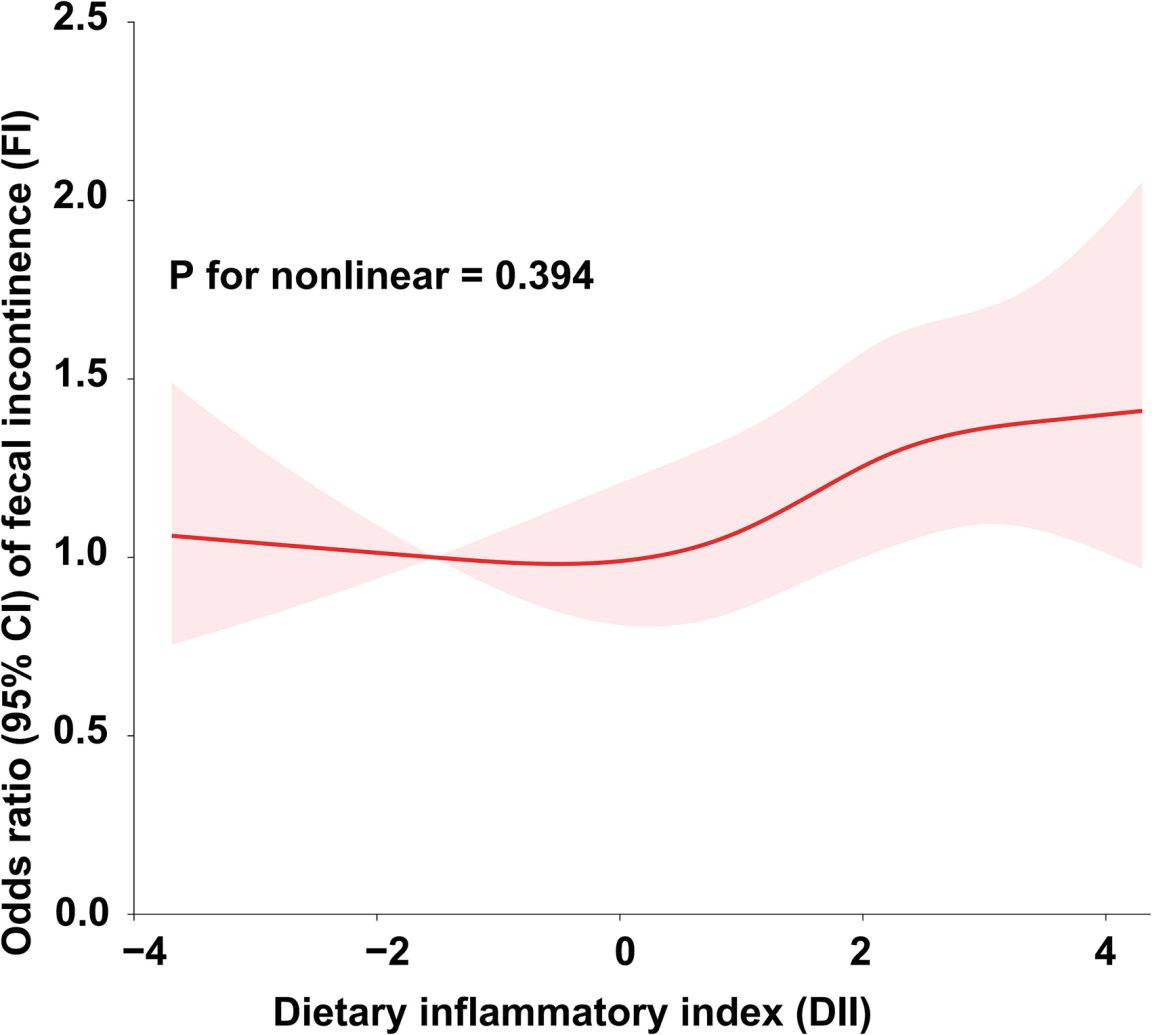
Association between DII and the risk of FI.

To further explore the relationship between DII and FI, we conducted weighted generalized logistic regression analysis. Our findings indicated that the greater DII was significantly linked with increased prevalence of FI in the non-adjusted model (OR = 1.08, 95% CI, 1.03–1.13, *p* = 0.0012), Model I (OR = 1.07, 95% CI, 1.02–1.12, *p* = 0.006), and Model II (OR = 1.05, 95% CI, 1.00–1.10, *p* = 0.0486) (Table 2). The non-adjusted model showed that the prevalence of FI increased by 8% for every unit rise in DII (OR = 1.08, 95% CI, 1.03–1.13, *p* = 0.0012). Furthermore, we divided DII into three equal pieces and transformed it from a continuous variable to a categorical variable. After comprehensive adjustment, our results showed that the prevalence of FI increased statistically significantly to 27% in participants in the highest DII tertile (DII-T3) as opposed to the lowest DII tertile (DII-T1) (OR = 1.27, 95% CI, 1.06–1.53, *p* = 0.0113). Additionally, we employed restricted cubic spline (RCS) regression to depict the connection between DII and FI. Overall, the RCS suggests a nonlinear positive correlation between DII and FI (Figure 2).

**Table 2:** Weighted logistic regression analysis on the association between DII and FI.

	Non-adjusted model		Model I		Model II	
	OR [95% CI]	*P*-value	OR [95% CI]	*P*-value	OR [95% CI]	*P*-value
DII	1.08 (1.03, 1.13)	**0.0012**	1.07 (1.02, 1.12)	**0.0060**	1.05 (1.00, 1.10)	**0.0486**
DII-T1	Reference	–	Reference	–	Reference	–
DII-T2	1.20 (1.00, 1.44)	**0.0481**	1.21 (1.01, 1.45)	**0.0419**	1.18 (0.98, 1.42)	0.0791
DII-T3	1.40 (1.17, 1.67)	**0.0002**	1.35 (1.13, 1.62)	**0.0012**	1.27 (1.06, 1.53)	**0.0113**
P for trend	**<0.001**		**0.001**		**0.012**	

Data are presented as OR (95% CI). Model I was adjusted for gender, age, race. Model II was adjusted for gender, age, race, smoked, alcohol, BMI, hypertension, arthritis, liver disease, cancer, asthma. OR, odds ratio; CI, confidence interval; DII, dietary inflammation index; FI, Fecal incontinence. *P* values < 0.05 are shown in bold.

### The correlation between DII and FI is not affected by other indicated covariates

To address the issue of between-group heterogeneity and to assess whether the relationship between DII and FI prevalence was stable across demographic characteristics, we conducted subgroup analyses using fully adjusted logistic regression models for different covariates (gender, age, race, smoked, alcohol, BMI, hypertension, arthritis, liver disease, cancer, asthma). As shown in Table 3, including gender (male/female), age (20–40 year/41–60 year/≥60 year), race (Mexican American/Other Hispanic/Non-Hispanic White/Non-Hispanic Black/Other Race), smoking (yes/no), drinking (yes/no), BMI (Underweight/Normal weight/Overweight/Obese), hypertension (yes/no), arthritis (yes/no), liver disease (yes/no), cancer (yes/no), and asthma (yes/no), none of the covariates had a significant effect on the correlation between DII and FI (P for interaction > 0.05). In conclusion, the nonlinear positive correlation between DII and FI prevalence is not affected by potential bias due to other confounders, implies that a pro-inflammatory diet is indeed a causative factor for FI.

**Table 3:** Subgroup analyses according to gender, age, race, smoked, alcohol, BMI, hypertension, arthritis, liver disease, cancer, asthma.

Variables	Odds ratio	*P*-value	P for interaction
Gender, n (%)			0.5003
Male	1.03 (0.96, 1.11)	0.3513	
Female	1.07 (1.00, 1.14)	0.0605	
Age, n (%)			0.2454
20–40	1.15 (1.01, 1.29)	0.0302	
40–60	1.06 (0.97, 1.15)	0.1863	
≥60	1.02 (0.95, 1.09)	0.5744	
Race, n (%)			0.5497
Mexican American	1.08 (0.95, 1.24)	0.2506	
Other Hispanic	0.92 (0.77, 1.11)	0.3893	
Non-Hispanic White	1.07 (1.00, 1.14)	0.0347	
Non-Hispanic Black	1.00 (0.89, 1.13)	0.9639	
Other Race	1.09 (0.84, 1.43)	0.5141	
Smoked, n (%)			0.9759
Yes	1.05 (0.98, 1.12)	0.1445	
No	1.05 (0.98, 1.13)	0.1445	
Alcohol, n (%)			0.2574
Yes	1.07 (1.01, 1.13)	0.0235	
No	1.01 (0.92, 1.10)	0.9046	
BMI, n (%)			0.0769
Underweight	1.41 (0.96, 2.06)	0.0815	
Normal weight	0.99 (0.91, 1.08)	0.0815	
Overweight	1.12 (1.03, 1.22)	0.0093	
Obese	1.02 (0.94, 1.11)	0.5856	
Hypertension, n (%)			0.5388
Yes	1.07 (0.99, 1.15)	0.0751	
No	1.04 (0.97, 1.11)	0.2636	
Arthritis, n (%)			0.7011
Yes	1.06 (0.98, 1.15)	0.1167	
No	1.04 (0.98, 1.11)	0.1873	
Liver disease, n (%)			0.6839
Yes	1.11 (0.85, 1.45)	0.4470	
No	1.05 (1.00, 1.10)	0.0606	
Cancer, n (%)			0.5158
Yes	1.09 (0.97, 1.22)	0.1513	
No	1.04 (0.99, 1.10)	0.1226	
Asthma, n (%)			0.8513
Yes	1.04 (0.93, 1.17)	0.5043	
No	1.05 (1.00, 1.11)	0.5043	

Model II was used in subgroup analysis, which was adjusted for gender, age, race, smoked, alcohol, BMI, hypertension, arthritis, liver disease, cancer, asthma.

### Identifying key dietary factors using multiple machine learning algorithms

**Figure 3:**
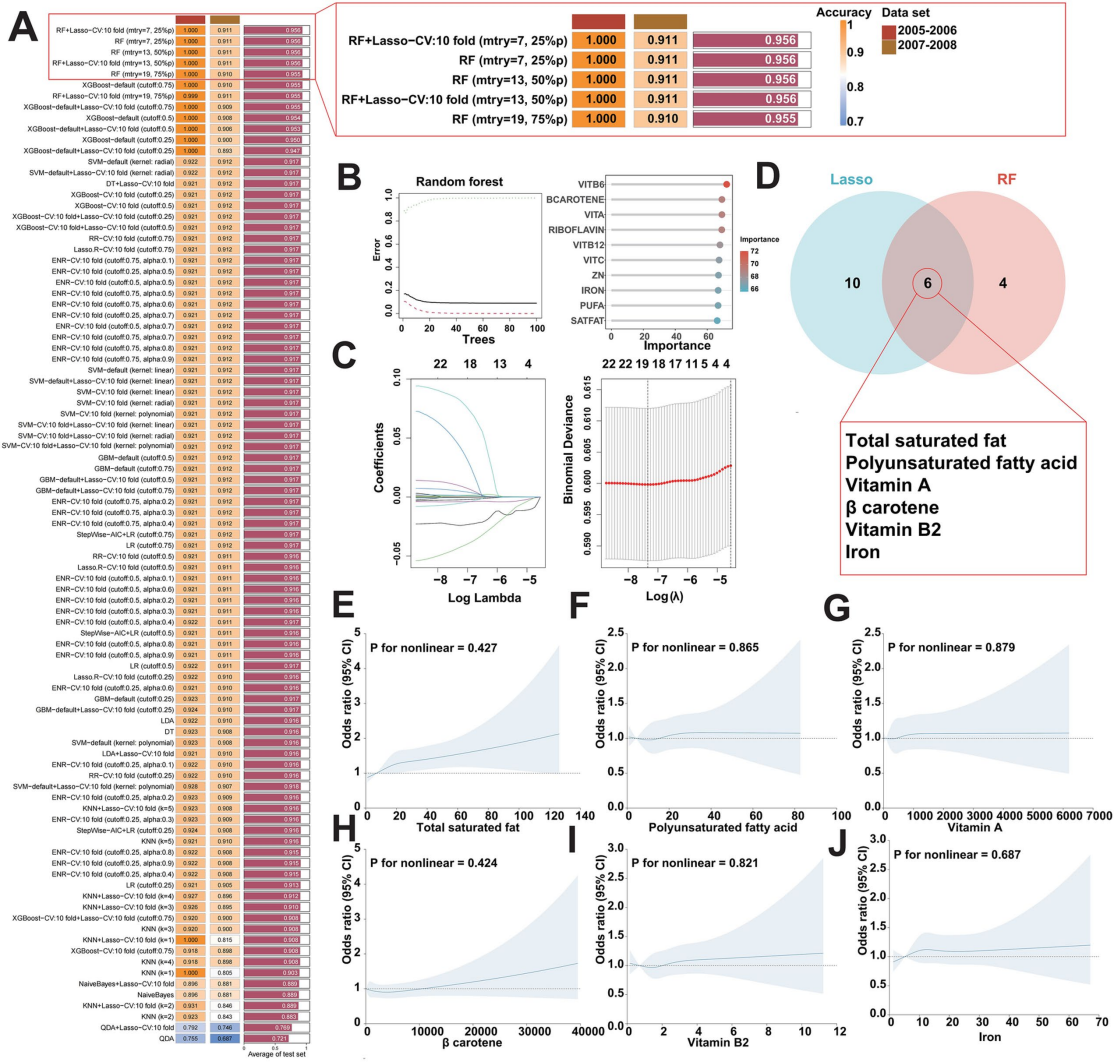
Identification of key dietary factors for FI using multiple machine learning algorithms. **(A)** Utilizing 93 combinations of machine learning algorithms to determine the best model for screening key dietary factors and calculate the C index for each model in two cycle datasets (2005–2006, 2007–2008) in the NHANES database. **(B,C)** Visualization of RF in two cycle datasets (2005–2006, 2007–2008) in the NHANES database **(B)** and visualization of Lasso regression **(C)**. **(D)** Lasso and RF algorithms work together to identify key dietary factors for FI. **(E–J)** Weighted restricted cubic spline curve describing the dose–response relationship between the key dietary factors and FI risk. Total saturated fat intake **(E)**; polyunsaturated fatty acid intake **(F)**; vitamin A intake **(G)**; *β* carotene intake **(H)**; vitamin B2 intake **(I)**; iron intake **(J)**.

Next, to identify the key dietary factors that have the greatest contribution to FI among the 25 dietary components included in DII, we applied 13 different machine learning algorithms, including LR, LDA, QDA, KNN, DT, RF, XGBoost, RR, Lasso, ENR, SVM, GBM, and NaiveBayes. These algorithms were combined in a strategic manner through the use of a 10-fold cross-validation methodology, with the assistance of a C-index performance metric to determine the combination that yielded the most optimal results. This iterative process was performed meticulously on two cycle datasets (2005–2006, 2007–2008) in the NHANES database, as illustrated in Figure 3A. Of the 93 models studied, five predictive models all comprised with Lasso and/or RF showed the highest average C-index, which demonstrated satisfactory performance in the aforementioned both two cycle datasets. In general, Lasso and RF combined model emerged as the most promising model, characterized by high accuracy. Notably, the RF algorithm selected out 10 potential key dietary factors (Figure 3B), while the Lasso algorithm screened 16 candidates (Figure 3C). Consequently, the synergistic integration of the Lasso and the RF algorithm led to the identification of 6 key dietary factors, including total saturated fat, polyunsaturated fatty acid, vitamin A, *β* carotene, vitamin B2, and iron (Figure 3D). Subsequently, we analyzed the association between the intake of total saturated fat, polyunsaturated fatty acid, vitamin A, β carotene, vitamin B2, or iron and the risk of FI. Multivariate adjusted RCS curves showed the risk of FI was increasing with promoted intake of the indicated key dietary factors (Figures 3E–J), suggesting the pivotal roles of these key dietary factors in contributing to the development of FI.

### Developing a risk prediction model for FI based on key dietary factors

**Figure 4:**
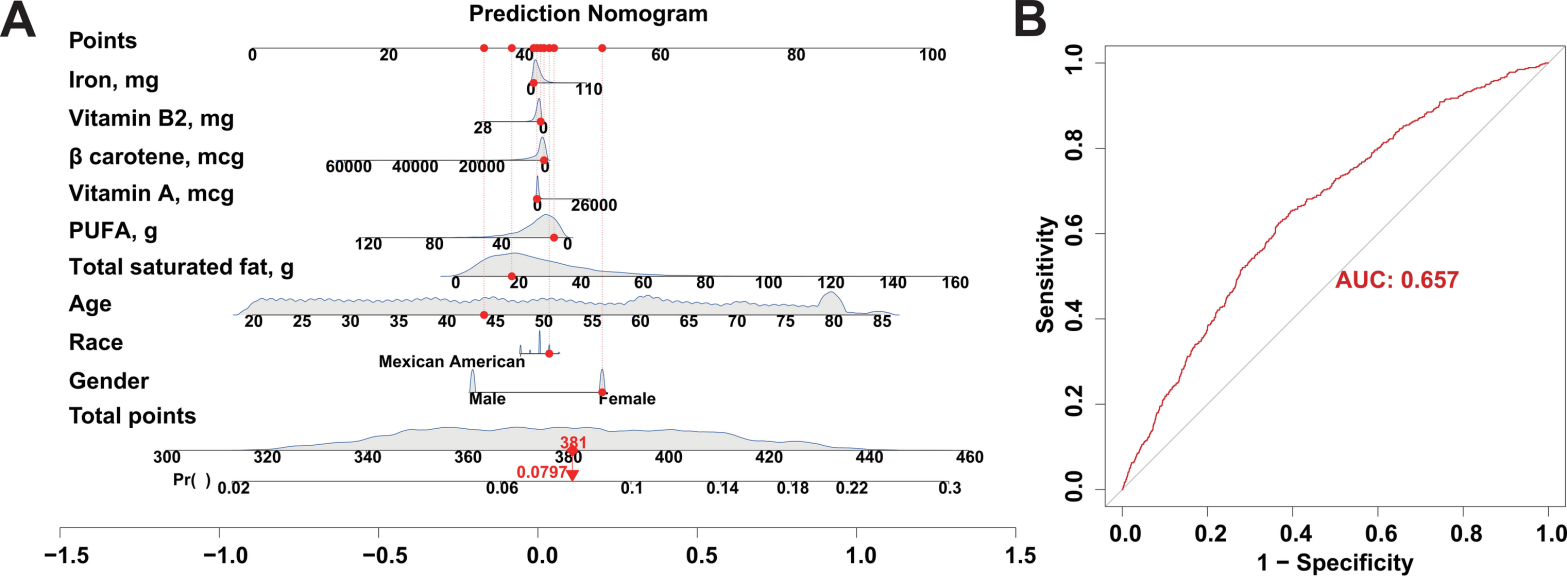
Construction of a risk prediction model for FI using participants from the NHANES database between 2005 and 2008 as a training cohort. **(A)** Nomogram model based on age, gender, and ethnicity and the 6 key dietary factors for FI. **(B)** ROC curves used to assess the FI predictive performance of the nomogram model.

As the key dietary factors are dramatically positively correlates with the risk of FI, we sought to construct an adjunct tool for FI diagnosing based on these dietary intakes. A risk prediction model for FI was constructed based on participants retrieved from the NHANES database in 2005–2008 by incorporating the basic population characteristics (age, sex, race) and aforementioned key dietary factors (Figure 4A). The predictive performance of the risk model was evaluated by using ROC curves. Our result presents a good predictive capability of the risk model as the AUC score was 0.657 (Figure 4B). To further validate the accuracy of our risk model, we also enrolled an independent participant cohort retrieved from the NHANES database in 2009–2010. With the AUC score of 0.684 (Supplementary Figures S2A,B), our results again demonstrated the reliability and accuracy of the risk prediction model based on key dietary factors. Together, the effects of our novel developed FI risk prediction model based on the key dietary factors has been evaluated in nearly 10,000 participants enrolled in NHANES, suggesting its promising potential in the clinical diagnosis of FI.

### Intake of key dietary factors contributes to individuals’ inflammatory response

**Figure 5:**
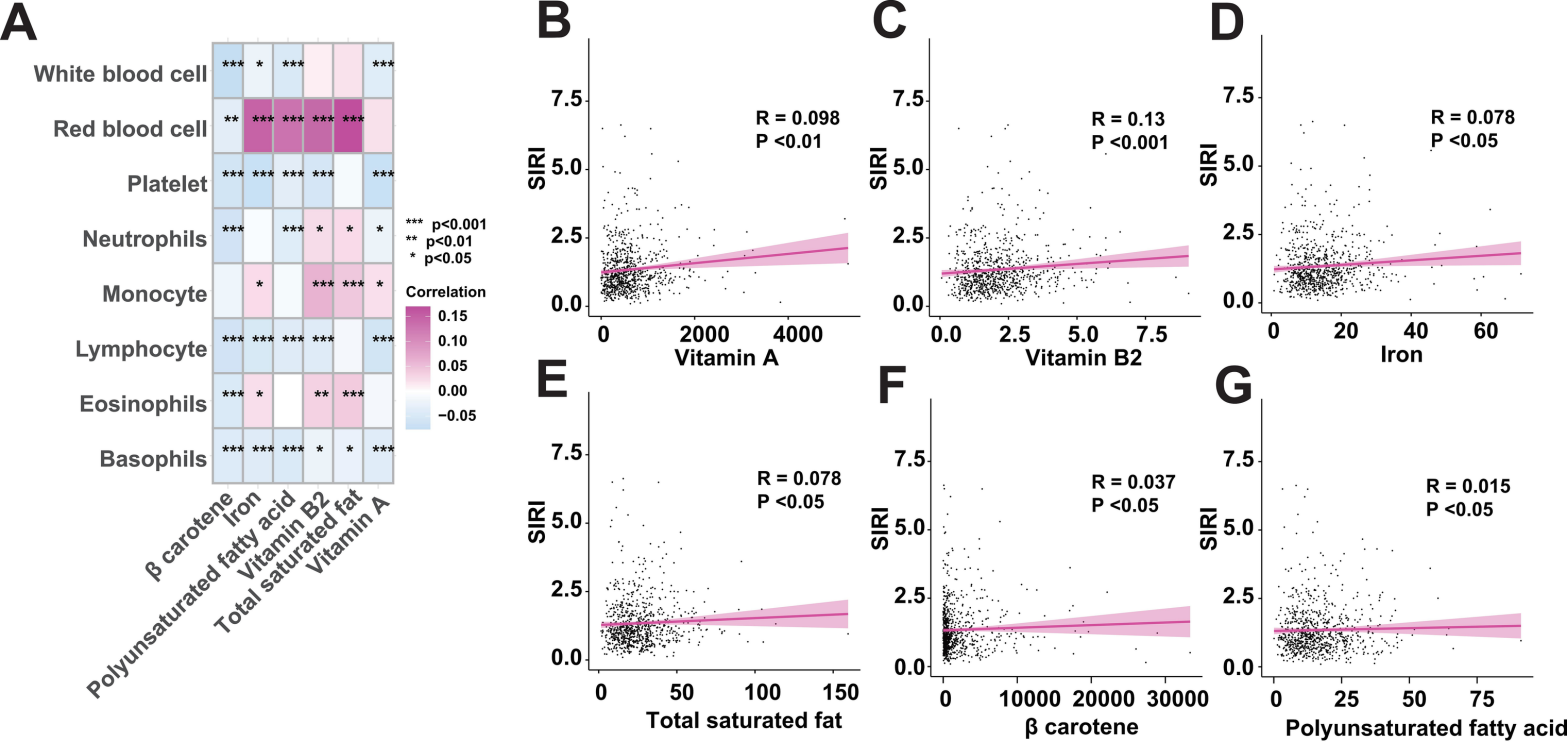
The intake of key dietary factors raises inflammatory levels. **(A)** Correlation analysis between the key dietary factors and inflammatory indicators. **(B–G)** Intake of key dietary factors is positively associated with systemic inflammation response index (SIRI).

Although the above findings have revealed the contribution of total saturated fat, polyunsaturated fatty acid, vitamin A, *β* carotene, vitamin B2, and iron, named as key dietary factors, to the increasing risk of FI, the detailed roles of these key dietary factors as pro-inflammatory diets leading to an internal inflammatory response that in turn promotes the development of FI remains unknown. To address this, we firstly included inflammatory cell analysis to investigate whether the intake of the identified key dietary factors activates the inflammatory response *in vivo* (Supplementary Table S2). Through correlation analysis, we found that the intake of the identified 6 key dietary factors was significantly correlated with the altered levels of inflammatory cells for almost all the involved variables, including white blood cell, red blood cell, platelet, neutrophils, monocyte, lymphocyte, eosinophils, and basophils, which indicates that the intake of the key dietary factors may influencing an individuals’ inflammatory status (Figure 5A). Then, by measuring the systemic inflammation response index (SIRI), our results showed that the intake of the key dietary factors was positively associated with SIRI levels, suggesting the contributions of intaking total saturated fat, polyunsaturated fatty acid, vitamin A, *β* carotene, vitamin B2, and iron in inducing individuals’ inflammatory response (Figures 5B–G).

### Key dietary factors inducing FI though exacerbating intestinal inflammation

**Figure 6:**
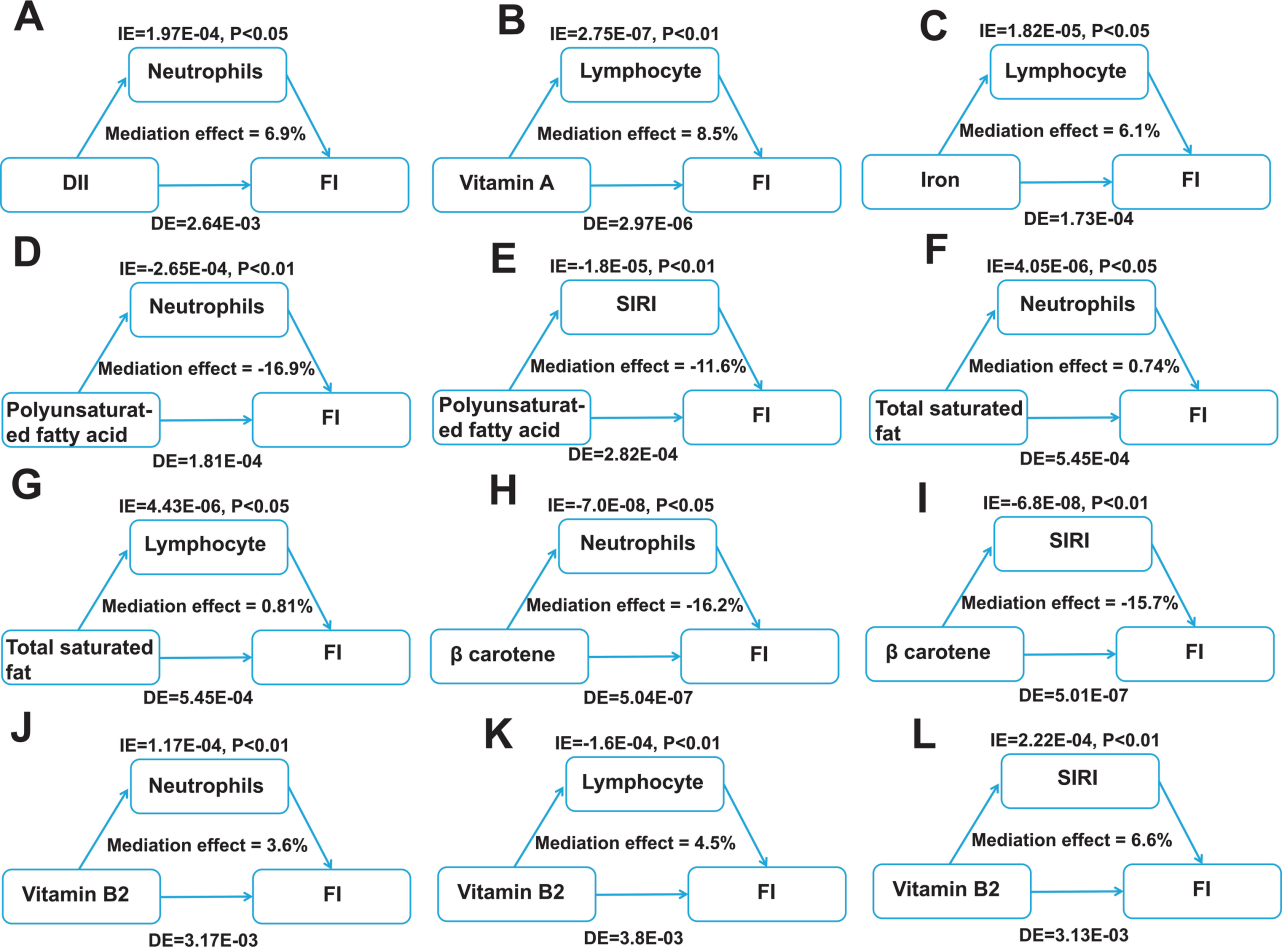
Identification of mediating factors between the key dietary factors and FI risk. **(A)** The mediating effects of neutrophils between DII and FI. **(B)** The mediating effects of lymphocyte between vitamin A and FI. **(C)** The mediating effects of lymphocyte between iron and FI. **(D–E)** The mediating effects of neutrophils **(D)** and SIRI **(E)** between polyunsaturated fatty acid and FI. **(F,G)** The mediating effects of neutrophils **(F)** and lymphocyte **(G)** between total saturated fat and FI. **(H,I)** The mediating effects of neutrophils **(H)** and SIRI **(I)** between β carotene and FI. **(J–L)** The mediating effects of neutrophils **(J)**, lymphocyte **(K)** and SIRI **(L)** between vitamin B2 and FI. DII, dietary inflammatory index; FI, Fecal incontinence; IE, the estimate of the indirect effect; DE, the estimate of the direct effect; SIRI, systemic inflammation response index.

Moreover, to further identifying the intermediary inflammatory factors in mediating one’s inflammatory response, we conducted mediation analysis and investigated the effect of inflammatory indicators between the key dietary intake and FI risk. As shown in Figure 6, we assessed the mediating roles of inflammatory indicators and visualized the significant correlated factors, including lymphocyte, neutrophils and SIRI. Our results suggest neutrophils and lymphocyte play pivotal mediating roles in regulating key dietary factors-induced FI.

**Figure 7:**
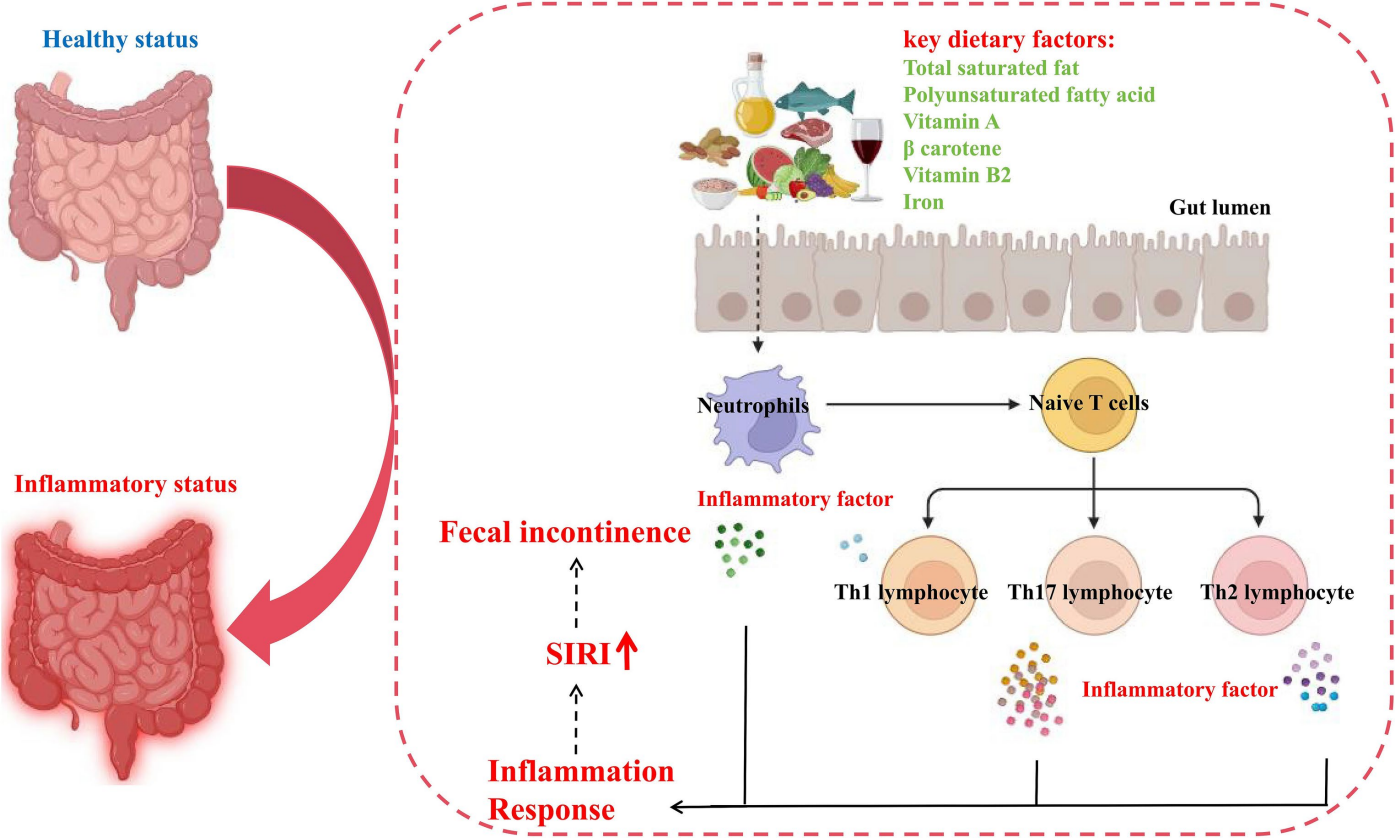
Potential pathomechanism of pro-inflammatory diet in inducing FI. SIRI, systemic inflammation response index.

Next, we investigated the potential mechanisms by which key dietary factors induce intestinal inflammation and ultimately affecting FI by integrating literature reports and signaling pathways analyses of inflammatory bowel disease annotated in KEGG (hsa05321). The aforementioned findings in our mediation analysis have suggested that the intake of key dietary factors promoting FI may be mediated by neutrophils and lymphocyte. When patients with intestinal inflammation consume a key dietary factor, the factor triggers an immune response in the body, i.e., neutrophils are activated and their abundance increases, which activates naive T cells, leading to the differentiation of these cells into Th1, Th2, and Th17, which then release a large number of inflammatory factors and activate a series of inflammatory responses in the body, exacerbating the development of FI (Figure 7). Overall, the intake of key dietary factors exacerbates intestinal inflammation, which in turn triggers the development of FI.

## Discussion

As various dietary cultures have taken root, individuals have developed diverse dietary patterns. Among these, pro-inflammatory diets have been linked to the prevalence of numerous intestinal disorders, including chronic diarrhea, functional constipation, crohn’s disease, and others (36–38, 57, 58). However, the associations between FI, a symptom that may be attributable to inflammatory intestinal disorders, and pro-inflammatory diets remains to be elucidated. It is noteworthy that the DII index developed by Shivappa et al. has the capacity to effectively assess the level of *in vivo* inflammatory changes resulting from dietary intake. This is demonstrated by the observation that a higher DII score is associated with a higher level of *in vivo* inflammation (28). Currently, the DII index has been employed to assess the relationship between several inflammatory diseases and diet (40, 41). In the present study, we collected the DII data of 31,034 participants from NHANES in order to explore the correlation between a pro-inflammatory diet and the incidence of FI. In particular, our analysis revealed that the older female population exhibited a high prevalence of FI. This finding aligns with previous observation, reinforcing the reliability of our analysis (59, 60). Additionally, our baseline analysis of the population showed that smoking, obesity, hypertension, arthritis, and asthma might also be contributing factors to FI. Notably, we also found a nonlinear positive correlation between DII and FI incidence following the adjustment for potential confounders using logistic regression analysis and the RCS analysis, suggesting that pro-inflammatory diet is an independent causative factor for FI, which provides the possibility of dietary applications in clinical diagnosis and assessment for FI.

Individuals affected with FI frequently experience feelings of embarrassment due to their inability to control bowel function (3). It has been shown that 45–50% of FI patients suffer from severe psychological disability (7). Severe social stigmatization often leads to significant underreporting of the condition, which further contributes to inadequate management (61). Worse, many individuals lack an understanding of FI and patients are often unable to describe their condition when not asked questions about the precise definition of FI. This concept-less awareness of FI leads to a substantial number of patients being delayed in treatment or never being treated, which exacerbates the severity of their FI and can even give rise to uncontrollable malignant complications. Therefore, there is a necessity to develop a more accessible FI prediction tool to facilitate the early diagnosis of FI, thereby enabling patients to be aware of having FI in a timely manner.

It is evident that machine learning has a distinct advantage in the development of clinical diagnostic models (62). Over recent decades, machine learning has evolved from a relatively peripheral technology to a cornerstone of healthcare data analytics, where it is highly effective in identifying key factors of disease, making accurate clinical diagnoses, and determining optimal treatment options in the biomedical field. Notably, machine learning has been extensively employed in the clinical management of intestinal diseases and been demonstrated to be effective. Previous research has shown that machine learning can improve the care of patients with inflammatory bowel disease at all stages of the disease process (63). Furthermore, clinicians can now utilize key factors identified by machine learning for early diagnosis of disease, enabling the implementation of timely interventions (64). Based on the aforementioned advantages of machine learning in inflammatory bowel disease, it is feasible to utilize 13 machine learning methods to screen out key dietary factors for FI in the present study.

Among the various algorithmic framework combinations, the Lasso and RF combined model emerged as the most effective model, characterized by high accuracy with minimal variables. Using the combined Lasso and RF model, we identified six key dietary factors, including total saturated fat, polyunsaturated fatty acid, vitamin A, *β* carotene, vitamin B2, and iron. Among them, polyunsaturated fatty acid is mainly found in sunflower, corn and soybean oils, and have been shown to influence inflammatory processes in chronic inflammatory disorders, such as aggravating Crohn’s disease by inducing lipid peroxidation in intestinal epithelial cells. In addition, total saturated fats, mainly from animal sources such as beef, lamb, pork, poultry and dairy products, in combination with cadmium can lead to macrophage M1 polarization, which promotes inflammatory activation and leads to chronic inflammation *in vivo* (30). Inflammatory illnesses are becoming more common due in part to deficiencies in certain micronutrients. For instance, vitamin A, which is enriched in animal liver, controls the conversion of T effector cells, thereby altering inflammatory and immune responses (32). β-carotene is mainly found in root vegetables and fruits. It has been shown that β-carotene can control the expression and production of inflammatory mediators such as TNF-*α* and IL-1β in a dose-dependent manner (65). Vitamin B2 is mainly derived from microbial fermentation and chemical synthesis. Low vitamin B2 reduces cell proliferation and accelerates apoptosis of immune cells, leading to immune system disorders (66). The main daily sources of iron are animal offal, green leafy vegetables and grains. Iron accumulation in monocytes and macrophages has been shown to closely correlated with inflammatory factors inducing (67). However, as aforementioned, the pro-inflammatory or anti-inflammatory properties of nutrients are not completely absolute, and are related to multiple factors including the dose-dependent effect, external environment, host characteristics and others (33–35). While these micronutrients exert pro-inflammatory effects in a certain condition, their contextual interactions with dietary matrices, host microbiota, and environmental cofactors may invert these functions. Our population-level associations should be interpreted as indicators of complex diet-host interactions rather than intrinsic nutrient properties. Nevertheless, the involvement of these dietary components in managing the inflammatory state of various diseases supports their pivotal potential in evaluating the risks of FI.

Therefore, based on the indicated key dietary factors and the basic population characteristics, we constructed a risk prediction model for FI. The relative high AUC score validated in both train and validation cohorts of NHANES (2005–2008, 2009–2010), including nearly 10,000 participants, indicated that the risk model performs well in FI diagnosis and self-evaluation. A more convenient application of the risk model is to assist those who lack conceptual awareness of FI. Individuals only need to input their age, gender, race, and daily intake of the aforementioned six key dietary components to obtain their risk of FI from the nomogram. Consequently, the risk model developed in the present work might facilitate the early diagnosis and evaluation of FI in a user-friendly manner, thereby enabling timely medical interventions to be taken.

As elucidated in our study, pro-inflammatory diets are highly associated with FI, and can predict the risks for developing FI. To further decipher the biological mechanisms driving these associations, we introduced a mediator analysis of inflammatory indicators as pro-inflammatory diets are thought to be an important contributor to intestinal inflammation, which in turn is a well-documented cause of FI. In the present study, our results suggest that neutrophils and lymphocytes are the main mediators of inflammatory response and FI. Neutrophils are a key class of leukocytes that play an important role in the innate immune response. These cells are equipped with multiple signaling pathways including presentation, phagocytosis and release of inflammatory factors (68). Lymphocytes, including T-cells, B-cells and natural killer cells, are central to the adaptive immune response. They are regulated by complex signaling pathways that modulate their development, activation and differentiation functions. The major signaling pathway involved in lymphocyte function is the TCR pathway. Following antigen recognition, the TCR binds to peptides presented by the major histocompatibility complex (MHC) on antigen-presenting cells. This interaction triggers a cascade response involving the release of multiple inflammatory factors (69). In the present study, we found that neutrophils and lymphocytes mediated a proportional association between pro-inflammatory diet and FI by mediation analysis. Our findings confirmed the important mediating role of inflammation and provide valid evidence for its involvement in this association.

The present study still has certain limitations that need to be clarified. Firstly, the definition of FI is a complex process that is best assessed comprehensively using multiple methods. Secondly, although the present study fully adjusted for confounders, due to data limitations and missing details of some of the covariates in the NHANES database, which resulted in us still not being able to adjust for the entire covariates. Finally, the present study’s assessment of DII relied on the dietary recall, which may have overlooked chronic eating habits and introduced recall bias.

## Conclusion

In conclusion, the present study provides evidence that pro-inflammatory diet is positively associated with the risk of fecal incontinence, and also emphasizes the important mediating role of inflammation in this relationship. Moreover, the risk prediction model developed in the present study based on the pro-inflammatory diet can facilitate self-assessment and diagnosis in a more convenient manner, which may provide valuable assistance in the clinical evaluation of fecal incontinence.

## Data Availability

The original contributions presented in the study are included in the article/Supplementary material, further inquiries can be directed to the corresponding author.

## Glossary

FI: Fecal incontinence
NHANES: National Health and Nutrition Examination Survey
ROC: Receiver operating characteristic
RCS: Restricted cubic spline
SIRI: systemic inflammation response index
AUC: Area under curve
LR: Logistic Regression
LDA: Linear discriminant analysis
QDA: Quadratic discriminant analysis
KNN: k-Nearest Neighbor
DT: Decision tree
RF: Random forest
XGBoost: Extreme Gradient Boosting
RR: ridge regression
Lasso: Lasso regression
ENR: Elastic Net regression
SVM: Support vector machine
GBM: Grandient Boosting Machine
NaiveBayes: Naive Bayesian algorithm
SD: Standard deviations
OR: odds ratios
CI: Confidence intervals
