# Supplementary material for: Association between pro-inflammatory diet and fecal incontinence: a large population-based study
Source: Front Nutr. 2025 May 22;12:1547406. doi: 10.3389/fnut.2025.1547406 (PMC12137088; doi:10.3389/fnut.2025.1547406)
Supplement: Supplementary file 1 [file Table_1.pdf]

Table S1. Dietary intake of each DII component according to dietary inflammatory index tertiles.

Variables	DII-T1 (−4.54 to 0.79, n = 3126)	DII-T2 (0.79 to 2.22, n = 3125)	DII-T3 (2.22 to 4.49, n = 3126)	P-value
**DII**	-0.46 ± 0.96	1.53 ± 0.41	2.97 ± 0.50	**<0.001**
**Energy, kcal**	2630.55 ± 1088.57	2137.55 ± 830.29	1640.12 ± 727.36	**<0.001**
**Protein, g**	103.07 ± 46.47	81.21 ± 36.05	59.14 ± 29.25	**<0.001**
**Carbohydrate, g**	321.46 ± 135.89	257.34 ± 107.42	203.44 ± 100.35	**<0.001**
**Dietary fiber, g**	23.52 ± 11.27	15.07 ± 7.09	9.66 ± 4.95	**<0.001**
**Total fat, g**	97.26 ± 52.72	80.48 ± 39.99	62.34 ± 34.37	**<0.001**
**Total saturated fat, g**	31.22 ± 19.42	26.60 ± 14.94	21.09 ± 13.01	**<0.001**
**MUFA, g**	35.64 ± 20.70	29.60 ± 15.70	23.00 ± 13.48	**<0.001**
**PUFA, g**	21.87 ± 12.77	17.22 ± 10.07	12.74 ± 8.23	**<0.001**
**Cholesterol, mg**	347.90 ± 279.93	300.49 ± 232.05	235.38 ± 187.79	**<0.001**
**Vitamin E, mg**	0.89 ± 3.53	0.30 ± 2.08	0.10 ± 0.82	**<0.001**
**Vitamin A, mcg**	915.80 ± 809.57	548.68 ± 614.52	343.04 ± 322.47	**<0.001**
**β carotene, mcg**	3526.29 ± 5185.41	1548.92 ± 2313.16	753.75 ± 1230.80	**<0.001**
**Vitamin B1, mg**	2.15 ± 1.08	1.57 ± 0.75	1.11 ± 0.55	**<0.001**
**Vitamin B2, mg**	2.80 ± 1.48	2.06 ± 1.03	1.50 ± 0.87	**<0.001**
**Niacin, mg**	32.33 ± 16.10	24.36 ± 12.55	17.35 ± 10.09	**<0.001**
**Vitamin B6, mg**	2.79 ± 1.48	1.92 ± 1.12	1.26 ± 1.03	**<0.001**
**Total folate, mcg**	261.48 ± 229.90	175.88 ± 151.07	119.07 ± 91.29	**<0.001**
**Vitamin B12, mcg**	7.08 ± 7.81	5.24 ± 7.03	3.50 ± 3.61	**<0.001**
**Vitamin C, mg**	131.13 ± 119.18	82.39 ± 95.73	47.42 ± 60.44	**<0.001**
**Magnesium, mg**	400.18 ± 153.61	279.14 ± 99.83	190.45 ± 76.98	**<0.001**
**Iron, mg**	20.53 ± 9.95	14.64 ± 6.69	10.21 ± 5.07	**<0.001**
**Zinc, mg**	15.47 ± 9.14	11.67 ± 8.60	8.13 ± 5.80	**<0.001**
**Selenium, mcg**	139.10 ± 70.62	110.51 ± 53.83	80.41 ± 41.39	**<0.001**
**Caffeine, mg**	168.42 ± 222.04	161.57 ± 209.65	165.19 ± 242.08	0.484
**Alcohol, g**	13.81 ± 29.96	11.77 ± 30.26	6.36 ± 22.93	**<0.001**

Data of dietary intake of DII components are presented as weighted mean [95% CI]. DII, dietary inflammatory index; MUFA, monounsaturated fatty acid; PUFA, polyunsaturated fatty acid.

Table S2. Inflammatory indicators of participants according to dietary inflammatory index tertiles.

Variables	DII-T1 (−4.54 to 0.79, n = 3126)	DII-T2 (0.79 to 2.22, n = 3125)	DII-T3 (2.22 to 4.49, n = 3126)	P-value
**White blood cell, (1000 cells/ μ L)**	7.01 ± 2.11	7.24 ± 2.26	7.49 ± 3.07	**<0.001**
**Lymphocyte, (1000 cells/ μ L)**	2.06 ± 0.79	2.16 ± 1.05	2.21 ± 1.01	**<0.001**
**Monocyte, (1000 cells/ μ L)**	0.55 ± 0.18	0.55 ± 0.19	0.55 ± 0.20	0.732
**Neutrophils, (1000 cells/ μ L)**	4.15 ± 1.65	4.28 ± 1.70	4.47 ± 2.52	**<0.001**
**Eosinophils, (1000 cells/ μ L)**	0.20 ± 0.18	0.21 ± 0.17	0.21 ± 0.23	**0.033**
**Basophils, (1000 cells/ μ L)**	0.03 ± 0.06	0.04 ± 0.06	0.04 ± 0.10	**<0.001**
**Red blood cell, (1000 cells/ μ L)**	4.71 ± 0.49	4.67 ± 0.50	4.61 ± 0.51	**<0.001**
**Platelet, (1000 cells/ μ L)**	249.82 ± 65.63	257.00 ± 67.98	265.10 ± 71.58	**<0.001**

Data of inflammatory factors are presented as weighted mean [95% CI].
