# Supplementary material for: Association between pro-inflammatory diet and fecal incontinence: a large population-based study
Source: Front Nutr. 2025 May 22;12:1547406. doi: 10.3389/fnut.2025.1547406 (PMC12137088; doi:10.3389/fnut.2025.1547406)
Supplement: Supplementary file 2 [file Image_1.pdf]

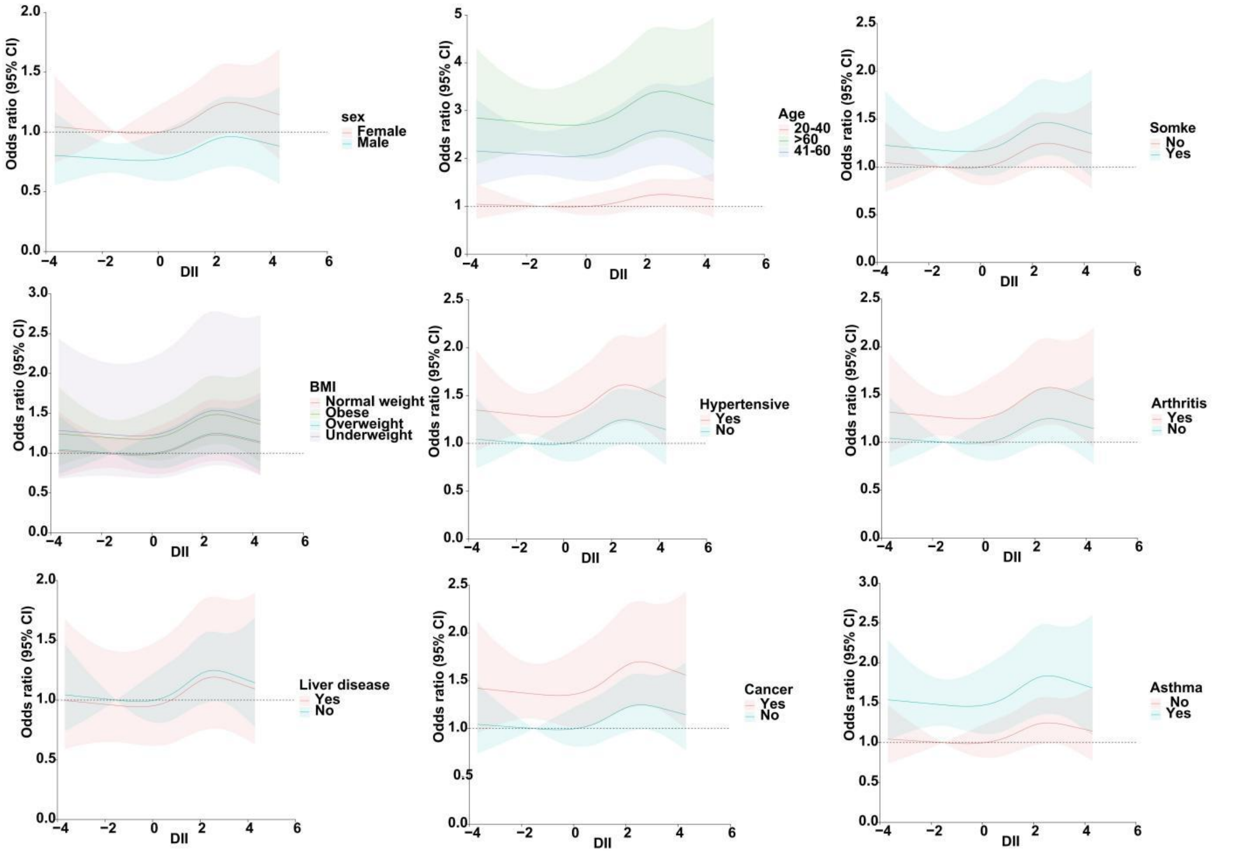

Figure S1. The RCS curves of the association between DII and FI among subgroups, respectively. RCS, restricted cubic spline; DII, dietary inflammatory index; BMI, body mass index.

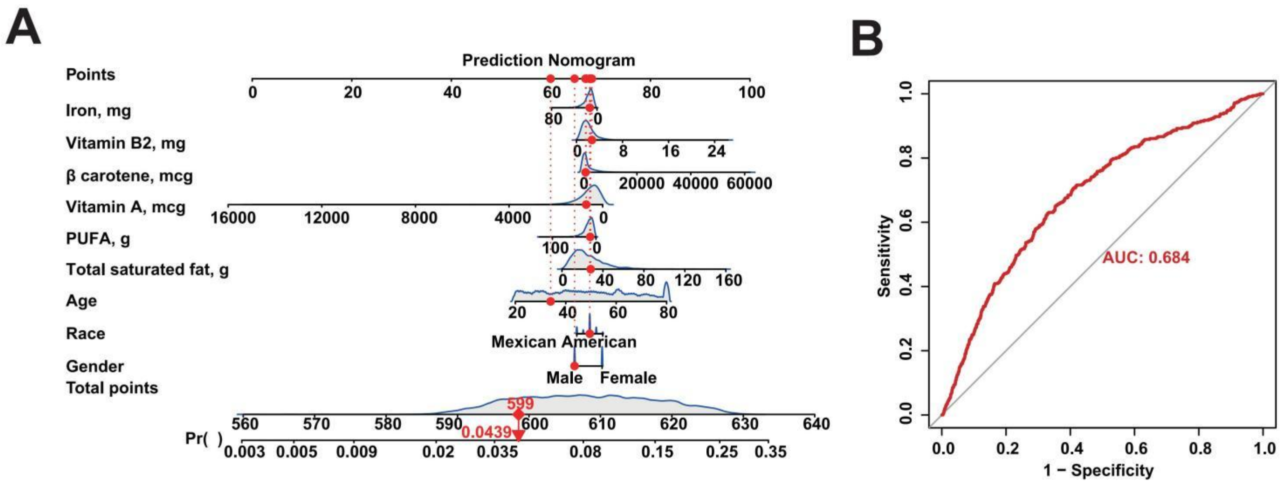

Figure S2. Construction of a risk prediction model for FI using participants from the nhanes database between 2009-2010 as a validation cohort. (A) Nomogram model based on age, gender, and ethnicity and the 6 key dietary factors for FI. **(B)** ROC curves used to assess the FI predictive performance of the nomogram model of validation cohort.
